# Bile duct diameter changes after laparoscopic cholecystectomy: a magnetic resonance cholangiopancreatography prospective study

**DOI:** 10.3325/cmj.2020.61.239

**Published:** 2020-06

**Authors:** Tomislav Pavlović, Sanja Trtica, Rosana Troskot Perić

**Affiliations:** 1 Faculty of Medicine, Josip Juraj Strossmayer University of Osijek, Osijek, Croatia; 2 Department of Radiology, St. Catherine Specialty Hospital, Zabok, Croatia; 3 Department of Radiology, University Hospital “Sveti Duh,” Zagreb, Croatia; 4 Department of Internal Medicine, University Hospital “Sveti Duh,” Zagreb, Croatia; 5 Faculty of Health Studies, University of Rijeka, Rijeka, Croatia

## Abstract

**Aim:**

To assess the intrahepatic and extrahepatic bile duct diameter dilatation after laparoscopic cholecystectomy with magnetic resonance cholangiopancreatography.

**Methods:**

Forty-eight patients (35 women, mean age 54.58 ± 11.83 years) underwent laparoscopic cholecystectomy because of gallstones. The intrahepatic and extrahepatic bile ducts were measured before and three and six months after cholecystectomy. The diameter was measured in the anteroposterior and laterolateral direction at 14 points.

**Results:**

When compared with the preoperative diameter, the common bile duct diameter at the proximal part was significantly wider three months (*P* = 0.006) and six months (*P* = 0.0001) after cholecystectomy; the common hepatic duct was significantly wider three months (*P* = 0.001) and six months (*P* = 0.003) after cholecystectomy; the right and left hepatic bile ducts were significantly wider six months after cholecystectomy (*P* < 0.0001, *P* = 0.01, respectively); and the segmental intrahepatic bile ducts in both hepatic lobes were significantly wider three months (*P* < 0.0001) and six months after cholecystectomy (*P* < 0.0001).

**Conclusion:**

This study showed that significant post-cholecystectomy dilatation occurred only at certain points and not along the whole extrahepatic bile duct. We also found a significant dilatation of the main intrahepatic and segmental intrahepatic bile ducts.

The common bile duct (CBD) dilatation occurs due to obstructive changes such as gallbladder tumor or pancreas tumor, choledocholithiasis, previous surgical procedures, and periampular diverticul (1-3). Additional causes may be cholecystectomy (4-9), age (4,10-12), and certain medications (13). However, some ultrasound (US) studies reported no dilatation after cholecystectomy (14-16) and one study reported dilatation after cholecystectomy in patients older than 60 years but not in younger patients (17). An endoscopic retrograde cholangiopancreatography (ERCP) study found CBD to be oval-shaped, meaning that the CBD diameter measured in the anteroposterior direction differed from the diameter measured in the laterolateral direction (18).

CBD diameter changes after cholecystectomy were observed with US, computerized tomography (CT), and ERCP, but most of the studies on this issue were retrospective and performed by US or CT. Due to its noninvasiveness, availability, and lower costs, US is the most frequently used technique for the analysis of bile ducts (19). It is a subjective, observer-dependent method, limited in the cases of colon meteorism or obesity. CT scan is also frequently used, although it can detect the CBD only in 30%-68% of patients (20-22). US and CT scan techniques do not register the juncture of the cystic duct and common hepatic duct, meaning that they do not differentiate between the common hepatic duct and common bile duct (CBD), which is why both ducts together are called the CBD. In patients with suspected cholelithiasis, ERCP has a sensitivity of 0.90 and a specificity of 0.95 (23).

Magnetic resonance cholangiopancreatography (MRCP) is a non-invasive “gold standard” for the evaluation of gallbladder and CBD pathology used to analyze both extrahepatic and intrahepatic bile ducts (24,25). So far, MRCP has been used only in the detection of choledocholithiasis and consequent dilatation, but not in the analysis of post-cholecystectomy dilatation. MRCP is close to the ideal diagnostic modality when it is based on correct clinical suspicion and predictive evaluation (26,27).

Considering the earlier controversial results on the change in the extrahepatic bile ducts diameter after cholecystectomy and the lack of previous research on post-cholecystectomy intrahepatic bile duct changes, this prospective MRCP study aimed to determine the extrahepatic and intrahepatic bile ducts diameter in the anteroposterior and laterolateral direction before and three and six months after laparoscopic cholecystectomy. We also aimed to resolve the controversies around the upper normal diameter limit of the bile ducts in MRCP examination in post-cholecystectomy patients.

## PATIENTS AND METHODS

This prospective study involved 50 patients older than 18 years who were referred for elective laparoscopic cholecystectomy because of cholelithiasis or cholesterolosis in the Department of Surgery University Hospital “Sveti Duh” in Zagreb from March 2017 till July 2018. The exclusion criteria were liver diseases (congenital disorders, metabolic disorders, cirrhosis, autoimmune diseases, infections, hepatitis, malignancies), pancreatic diseases (congenital disorders, autoimmune disorders, inflammatory disorders, malignancies), gallbladder and bile ducts disorders (acute inflammation of the gall bladder, acute inflammation of the bile ducts, choledocholithiasis), conversion of laparoscopic surgery to open cholecystectomy, and taking of glucagon, opioids, calcium blockers, atropine, progesterone, histamine_2_- receptor stimulators, theophylline, octreotide acetate, indomethacin, or erythromycin within three days before surgery. Patients with fat liver infiltration and benign liver tumor changes such as adenoma, hemangioma, and focal nodular hyperplasia were not excluded from the study. Patients were also not excluded from if more than six weeks passed from the end of the treatment of acute gall bladder inflammation until surgery and if they had regular laboratory tests that did not detect inflammation (leukocyte, blood differential test, C-reactive protein). One patient was excluded due to choledocholithiasis three months after surgery and one due to intrahepatic cholelithiasis six months after surgery. The final study group comprised of 48 patients (33 women).

**Figure 1:**
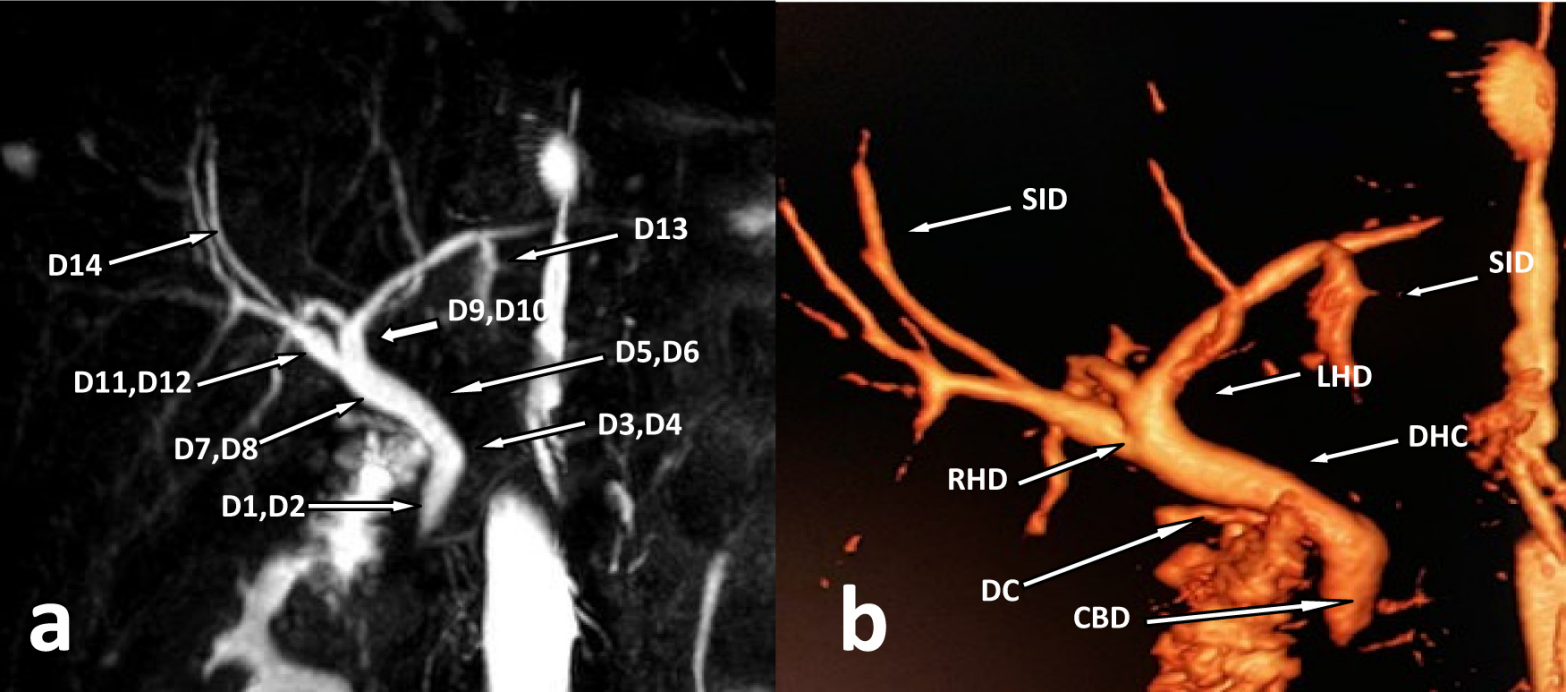
Magnetic resonance cholangiopancreatography (MRCP) of the intrahepatic and extrahepatic biliary tree (**A**) 3D MRCP – measurement points. (**B**) MRCP volume rendering technique – anatomy; CBD – common bile duct; DHC – common hepatic duct; DC – cystic duct; RHD – right hepatic bile duct; LHD – left hepatic bile duct; SID – segmental intrahepatic bile duct.

The study was performed in the Department of Radiology of the University hospital “Sveti Duh,” on MR device Phillips Achieva 1.5T (Phillips Medical Systems 2008, Eindhoven, the Netherlands). Patients underwent the examination after at least 8 hours of fasting, within 7 days before cholecystectomy, three months after cholecystectomy, and six months after cholecystectomy. The study was performed with body matrix coil in the MRCP protocol, using coronal three-dimensional high-resolution (3D MRCP HR) sequence with fat saturation: repetition time 1204 ms, echo time 650 ms, slice thickness 1.6 mm, and axial breath-hold balanced turbo field echo sequence: TR 3.7 ms, TE 1.8 ms, SL 5 mm. The examination covered the entire pancreaticobiliary tree. The common bile duct diameter was measured at two points in the distal part above the papilla Vateri and in the proximal part immediately below the connection of the cystic duct and the common hepatic duct. The common hepatic duct diameter was measured at two points in the distal part above the connection of the cystic duct and the common hepatic duct and in the proximal part. The left and right hepatic duct diameters were measured at a distance of 1 cm to their confluence (Figure 1). The diameters were measured in millimeters, rounded to one decimal, in the anteroposterior and laterolateral direction from the inner mucous layer to the inner mucous layer of the wall perpendicular to the longitudinal bile duct axis. In the left and right liver lobe, the widest segmental intrahepatic duct was measured in one dimension. The examination was processed at the same workstation by the same radiologist with eight years of experience.

In total, the intrahepatic and extrahepatic bile ducts diameters were analyzed at 14 points: D1 – diameter of the common bile duct in the distal part in the anteroposterior direction, D2 – diameter of the common bile duct in the distal part in the laterolateral direction, D3 – diameter of the common bile duct in the proximal part in the anteroposterior direction, D4 – diameter of the common bile duct in the proximal part in the laterolateral direction, D5 – diameter of the common hepatic duct in the distal part in the anteroposterior direction, D6 – diameter of the common hepatic duct in the distal part in the laterolateral direction, D7 – diameter of the common hepatic duct in the proximal part in the anteroposterior direction, D8 – diameter of the common hepatic duct in the proximal part in the laterolateral direction, D9 – diameter of the right hepatic duct in the anteroposterior direction, D10 – diameter of the right hepatic duct in the laterolateral direction, D11 – diameter of the left hepatic duct in the anteroposterior direction, D12 – diameter of the left hepatic duct in the laterolateral direction, D13 – diameter of the widest segmental intrahepatic duct in the right hepatic lobe, D14 – diameter of the widest segmental intrahepatic duct in the left hepatic lobe. The extrahepatic bile ducts were measured at eight points. The intrahepatic bile ducts were measured at six points, the right and left main hepatic bile duct at four points, and the segmental intrahepatic bile duct at one point in each hepatic lobe. The study was approved by the Ethics Committee of the University Hospital “Sveti Duh” (01-580).

### Statistical analysis

Categorical data are expressed as absolute and relative frequencies. The normality of distribution was tested with the Kolmogorov-Smirnov test. Numerical data are expressed as mean and standard deviation and median and interquartile range, where applicable. To assess the significance of differences in diameter before and after cholecystectomy, we used the repeated measures ANOVA test and Friedman ANOVA test, where applicable. In the *post-hoc* analysis, the Wilcoxon test and paired samples *t* test were used, where applicable. *Post-hoc* tests *P* values were Bonferroni corrected, and *P* < 0.0166 was considered as statistically significant. For the remaining results, the level of statistical significance was set at *P* < 0.05. The statistical analysis was conducted with MedCalc, version 16.2.0 (MedCalc Software bvba, Ostend, Belgium).

## RESULTS

**Figure 2:**
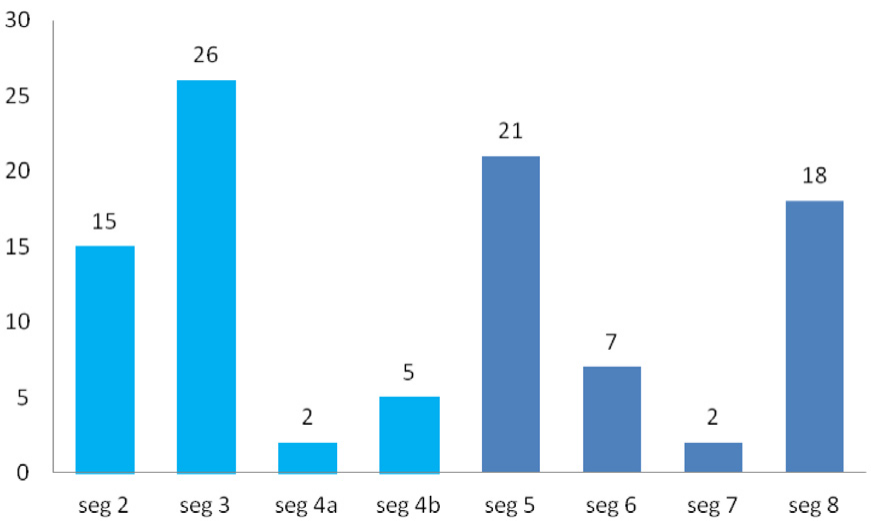
The most dilated segmental intrahepatic bile ducts in the liver segments (left liver lobe: segments 2, 3, 4a, 4b; right liver lobe: segments 5, 6, 7, 8).

The study comprised of 48 patients who underwent laparoscopic cholecystectomy, with a mean age of 54.58 ± 11.83 years (33 or 69% women). In all patients, gallstones were confirmed after surgery. Men and women did not significantly differ in mean age (men: 58.33 ± 11.36 years; women: 52.87 ± 11.81 years). After MRCP examination, no patients were diagnosed with Mirizzi syndrome. When compared with the preoperative diameter, the extrahepatic bile duct diameter at the points D1 (F = 2.67, df2 = 94, *P* = 0.07), D2 (F = 1.47, df2 = 94, *P* = 0.23), D5 (F = 2.35, df2 = 94, *P* = 0.10), and D6 (F = 2.13, df2 = 94, *P* = 0.12) did not change significantly after cholecystectomy. The extrahepatic bile duct diameter at the points D3, D4, and D8 was significantly wider three months after cholecystectomy and at the points D4, D7, and D8 was significatly wider six months after cholecystectomy (Table 1). The intrahepatic bile duct diameter at the points D10, D13, and D14 was significantly wider three months after cholecystectomy when compared with the preoperative diameter. The intrahepatic bile duct diameter at the points D11, D13, and D14 six months after cholecystectomy was significantly wider compared with the diameter three months after cholecystectomy. The intrahepatic bile duct diameter at the points D10, D11, D12, D13, and D14 six months after cholecystectomy was significantly wider compared with the preoperative diameter (Table 2). In the left hepatic lobe, the largest number of patients had the widest diameter of the segmental intrahepatic bile duct in the third segment and in the right hepatic lobe, in the fifth segment (Figure 2).

**Table 1:** Measurement points of the extrahepatic bile duct with significant diameter changes before and after cholecystectomy

	Bile duct diameter			p^¶^		
	M1	M2	M3	M2-M1	M3-M2	M3-M1
D3, mm	4.2 (3.60-5.30)	4.92 ± 1.16	5.0 (4.05-5.55)	0.006^**^	0.67^**^	0.03^**^
D4, mm	4.85 ± 1.21	5.50 ± 1.33	5.55 ± 1.28	<0.0001^‡^	0.99^‡^	0.0001^‡^
D7, mm	4.02 ± 1.22	4.20 (3.20-5.45)	4.59 ± 1.32	0.05^**^	0.35^**^	0.003^**^
D8, mm	4.25 (3.50-5.15)	5.02 ± 1.36	5.15 ± 1.49	0.001^**^	0.39^**^	<0.0001^**^

*M1 – the bile duct diameter before cholecystectomy; M2 – the bile duct diameter three months after cholecystectomy; M3 – the bile duct diameter six months after cholecystectomy; D3 – diameter of the common bile duct in the proximal part in the anteroposterior direction; D4 – diameter of the common bile duct in the proximal part in the laterolateral direction; D7 – diameter of the common hepatic duct in the proximal part in the anteroposterior direction; D8 – diameter of the common hepatic duct in the proximal part in the laterolateral direction.

†Data are expressed as mean ± standard deviation or median with interquartile range.

‡Repeated measures ANOVA test.

§Paired samples *t* test.

¶Bonferroni corrected.

**Wilcoxon test.

**Table 2:** The intrahepatic bile duct diameters measured before cholecystectomy, three months after cholecystectomy, and six months after cholecystectomy on six levels

	Bile duct diameter			*P*^¶^		
	M1	M2	M3	M2-M1	M3-M2	M3-M1
D9, mm	3.21 ± 0.75	3.30 (2.80-4.00)	3.40 (3.00-3.80)	0.02^**^	0.92^**^	0.04^**^
D10, mm	3.49 ± 0.72	4.00 ± 0.96	4.00 (3.60-4.50)	0.0001^§^	0.15^**^	<0.0001^**^
D11, mm	3.61 ± 0.62	3.66 ± 0.82	3.85 (3.25-4.55)	0.67^§^	0.003^**^	0.01^**^
D12, mm	3.49 ± 0.77	3.80 ± 0.88	3.86 ± 0.88	0.02^‡^	0.99^‡^	0.008^‡^
D13, mm	1.00 (0.90-1.20)	1.34 ± 0.35	1.70 (1.20-1.90)	<0.0001^**^	<0.0001^**^	<0.0001^**^
D14, mm	1.00 (0.95-1.15)	1.41 (1.10-1.65)	1.68 ± 0.43	<0.0001^**^	<0.0001^**^	<0.0001^**^

*M1 – the bile duct diameter before cholecystectomy; M2 – the bile duct diameter three months after cholecystectomy; M3 – the bile duct diameter six months after cholecystectomy; D9 – the diameter of right hepatic duct in the anteroposterior direction; D10 – the diameter of right hepatic duct in the laterolateral direction; D11 – the diameter of left hepatic duct in the anteroposterior direction; D12 – the diameter of left hepatic duct in the laterolateral direction; D13 – the diameter of the widest segmental intrahepatic duct in the right hepatic lobe; D14 – the diameter of the widest segmental intrahepatic duct in the left hepatic lobe.

†Data are expressed a mean ± standard deviation or median with interquartile range.

^‡^Repeated measures ANOVA test.

^§^Paired samples *t* test.

¶Bonferroni corrected.

^**^Wilcoxon test.

## DISCUSSION

This study showed that the intrahepatic bile duct significantly dilatated after cholecystectomy and that significant post-cholecystectomy dilatation occurred only at certain points along the extrahepatic bile ducts.

Different techniques produce different CBD diameter values. ERCP may show a larger CBD diameter due to increased pressures caused by the contrast medium injection in the CBD (28,29). CT measurements may be slightly larger (by 1.7 mm) than the US measurements because CT measurements are performed outer wall-outer wall, while US measurements are performed inner wall-inner wall (30,31). Most of the techniques (such as US and CT) cannot image the cystic duct because it is too small, so special care must be taken not to include the cystic duct in the CBD measurement (32). Our study has several advantages: the exclusion of patients who were taking medicines and had comorbidities that may affect the bile duct diameter; the intrahepatic and extrahepatic bile ducts diameter measurement in the frontal and sagittal planes at multiple points, and the high sensitivity and specificity of the MRCP examination, contributing to data accuracy. Three-dimensional approach used in this study achieves optimal visibility of the intrahepatic and extrahepatic bile ducts compared with 2D sequence (33). The rate of choledocholithiasis in our study was 1%, which is consistent with the study by Valkovic et al (7), who reported 2% of CBD stones after cholecystectomy. Previous studies have only analyzed extrahepatic bile ducts because most of the radiological methods do not detect intrahepatic bile ducts except if they are dilatated. However, MRCP is a non-invasive imaging modality that is able to analyze both the intrahepatic and extrahepatic bile ducts. By measuring the cross-section of the extrahepatic bile ducts, we established that they were oval-shaped, which is consistent with the study by Wachsberg et al (18). The CBD diameter did not significantly increase between the third and the sixth month, so we can conclude that the CBD was maximally dilatated three months after cholecystectomy. Many other studies also found post-cholecystectomy CBD dilatation (4,7-9,34-37). Most of these studies measured the CBD at 1-3 points, whereas our study used 8 measurement points. In addition, the previous studies were US, CT, or EUS-based, while our study used MRCP. The follow-up length in other studies was similar to ours and was mostly three, six, or twelve months, except in the study by Kaim et al (4), who followed their patients for 15 years. The upper limit of the normal extrahepatic bile duct diameter has been reported as 6 mm (9,38). In our study, at no point did the diameter exceed the upper limit of the normal diameter of 6 mm, although half of the measured points of the extrahepatic bile duct showed significantly wider diameter after cholecystectomy, which is consistent with the study by Feng (9).

No study so far has investigated the change of the intrahepatic bile duct diameter after cholecystectomy. We showed that, as opposed to the extrahepatic bile duct diameter, the intrahepatic bile duct diameter continues to dilatate after three months post-cholecystectomy. The upper limit of the normal intrahepatic bile duct diameter has been reported to be 2 mm (39). In our study, all the measured points of the segmental intrahepatic bile ducts showed significantly wider diameter after cholecystectomy but at no point did the diameter exceed the upper limit of 2 mm.

A limitation of our study is a short-term follow-up after cholecystectomy. Future studies should include a follow-up of at least two years or more to determine the long-term impact of cholecystectomy on the bile duct diameter changes. All the measurements were performed on the same machine by one experienced radiologist, so another limitation is the lack of inter-observer variability.

In conclusion, this study showed that post-cholecystectomy dilatation of the extrahepatic bile ducts only occurred at certain points. The common bile duct diameter significantly increased three months after cholecystectomy at the proximal part and remained increased six months after cholecystectomy, and the same happened with the common hepatic duct in the proximal part. We also found significant dilatation of the intrahepatic bile ducts. The upper limit of the normal extrahepatic bile ducts diameter was smaller than 6 mm and that of the intrahepatic segmental bile ducts was smaller than 2 mm, so only the values greater than these require further treatment and correlate with clinical and laboratory findings.

The unexpected dilatation of the extrahepatic bile ducts may present a treatment challenge. Knowing if cholecystectomy patients have a wider bile duct diameter compared with the general population can prevent unnecessary tests, especially costly procedures such as CT or ERCP examinations, which expose patients to radiation and potentially lead to complications.
